# A review of cutting-edge therapies for hepatocellular carcinoma (HCC): Perspectives from patents

**DOI:** 10.7150/ijms.59930

**Published:** 2021-06-18

**Authors:** Yan Gao, Liyang Lyu, Ye Feng, Fei Li, Yuanjia Hu

**Affiliations:** State Key Laboratory of Quality Research in Chinese Medicine, Institute of Chinese Medical Sciences, University of Macau, Taipa, Macao, China.

**Keywords:** ablation, trans artery chemoembolization (TACE), radiotherapy, T-cell, Notch pathway

## Abstract

**Rationale:** Hepatocellular carcinoma (HCC) is a challenging disease due to its heterogenous etiology. Several breakthroughs have occurred in treatment of HCC, associated with an enormous number of patent publications for a variety of HCC treatment modalities. As patents can provide valuable information for academic research and commercial development, this study aims to unravel the cutting-edge therapies for HCC by using patents as an indicator. The outcome from this analysis may offer meaningful insights for respective policymaking, strategic plan and research and development (R&D) prioritization.

**Methods:** Derwent Innovation platform was employed to collect the sample data of patents related to HCC treatment technologies worldwide as of December 31, 2019. Data inclusion, screening and exclusion were according to the rules of preferred reporting items for systematic reviews and meta-analyses (PRISMA). Technologies were classified based on Barcelona Clinic Liver Cancer (BCLC) staging system and recent clinical publications. Patent citation network analysis was carried out to identify and understand HCC therapeutic technology flow.

**Results:** A dataset of 2543 patent documents and 528 patent families was generated. 11 technological categories were classified. Numerous researches were focalized on refinements in technologies and innovations within the field of HCC therapy, and the major achievements are technology advancement on molecular target therapy, chemotherapy, locoregional therapy, combination therapy and immunotherapy with demonstrated clinical benefits. In patent citation network, Notch pathway investigation, antibody drug conjugate (ADC) technology development and drug eluting beads trans artery chemoembolization (DEB-TACE) advancement are the major technological communities involving patents with the greatest future exploratory potential.

**Conclusion:** Numerous emerging technologies have been identified in this study, in which exploring novel therapeutic targets in molecular target therapy, more localized and visible locoregional therapy and combination of immunotherapy with target therapy or other traditional therapies are highlighted as the future trends in treating HCC.

## Introduction

Liver cancer is the sixth most commonly diagnosed cancer and the fourth leading cause of cancer death globally 1. About 90% of liver cancer is hepatocellular carcinoma (HCC), which is the dominant type of primary liver cancer 2. HCC is typically associated with chronic liver injury - cirrhosis, because chronic hepatitis B virus (HBV) or hepatitis C virus (HCV) infection is the leading underlying cause of HCC 3. Moreover, there are multifarious risk factors for HCC including aflatoxins, obesity, alcohol, diabetes, anabolic steroids, hemochromatosis, nonalcoholic fatty liver disease etc. 4.

Given that HCC pathogenesis is complicated and heterogeneous, multidisciplinary team with diversified treatment modalities is essential to make decision by considering the tumor size, extent of tumor burden, functional status of the liver and patient performance status 5. Besides that, a staging system considering both the underlying liver disease and the HCC malignancy is important to determine the optimal therapy selection. Barcelona Clinic Liver Cancer (BCLC) staging system is such a system integrating both tumor and liver disease characteristics, and it is the most commonly used classification system for HCC 6. The BCLC staging system classifies disease severity into 5 stages: Stage 0 (very early stage), Stage A (early stage), Stage B (intermediate stage), Stage C (advanced stage), and Stage D (terminal stage), and the scale segregates patients into treatment recommendations specific to each stage 6. Resection, ablation, and liver transplant are considered as potent curative therapies, but only approximately 30% patients with early stage (BCLC stage 0 or A) HCC are eligible 6,7. At BCLC B (intermediate-stage disease), trans arterial chemoembolization (TACE) as a type of locoregional therapy is the only recommended option, but the side effects of TACE can limit the number of treatments 8. HCC patients at BCLC C (advanced-stage disease), for which local and curative therapies are not an option and the 5-year survival rates are only 18%, are recommended to be candidate for systemic therapy, by sorafenib or lenvatinib 9. However, this oral systemic treatment may not be appropriate for all patients due to its systemic toxicity leading to intolerance, potential patient compliance issues and applicability limited to patients with well-preserved liver function 9. At the last, symptomatic therapy is prescribed for end stage HCC (stage D) 6.

All in all, HCC is a challenging disease to treat due to its unique, complicated and heterogeneous etiology. Constantly, there is a medical unmet need of more effective and tolerated treatment options for diversified HCC characteristics. In the past years, there were many efforts on researching, developing and evaluating novel HCC therapeutic methods and technologies, which also led to abundant patent publications on HCC treatment technologies. These patents are worth noting not only as a legal shield of protecting interests of original researchers and developers by blocking competitors to copy innovations but in terms of the fact that new therapies usually publish patent documents before accessing to market. The implication of patents as an early signal of marketed products is emphasized in medical sector due to its lengthy, costly and risky process of R&D. However, patent documents represent a potentially useful but often underused resource 10. HCC treatment patents have not been systemically reviewed yet, while patent-related research on HCC treatment technology evolution is still lacking.

To address this knowledge gap, HCC therapeutic technologies were systemically reviewed from the perspective of patents by categorizing the technologies into distinctive groups mainly based on BCLC guideline and other relative clinical papers as well 6, 11-13. We chose to use patents as an indicator of cutting-edge therapies, because they present a crucial metric of today innovation, which could be developed into future products on market. In other words, patents are a cornerstone for the commercialization of a new field in life science and healthcare-related technologies. Moreover, patent analyses can be used to underpin important decision making and analyses by academics, industry, and governments. Looking onto international patent landscape could provide better understanding on the dynamics and activities of the invention ecosystem, as patent data is considered as result-based indicators of innovation and the reflection of technological development. This research is expected to show a comprehensive review of HCC therapies from patents perspective, which may provide an important overview and go-for reference for relevant decision making by clinicians, scientists, industrial partners, and policymakers.

## Milestones of HCC therapies

**Figure 1:**
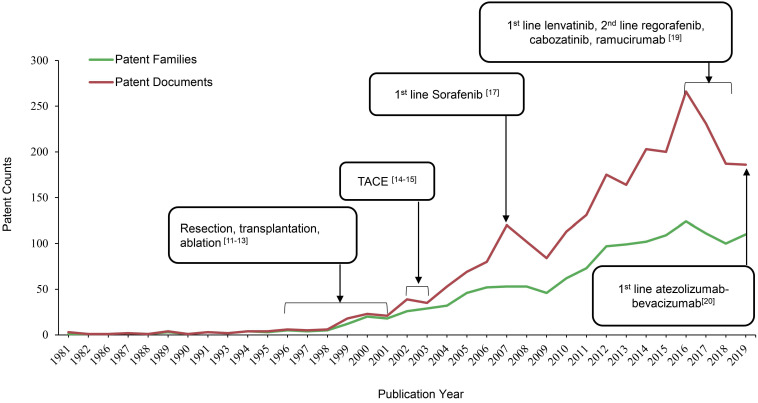
Milestones in HCC treatment aligned with patents publication trend.

As shown in Figure 1, the patent publication trend has a rising tendency over a period of 39 years, starting from 1 patent family (filed in 3 documents) in 1981 to 110 patent families (filed 186 documents) in 2019. Resection, transplantation and ablation have been established as curative therapy for early stage HCC by 1996-2001 14-16. 2002-03 was a turning point in the robust increase of HCC therapeutic patents, when trans artery chemoembolization (TACE) was established as the standard of care for intermediate stage of HCC 17,18. Since then, numerous researches were focalized on refinements in techniques and innovations of medical devices within this special issue, and the major achievements are radiation therapy, cytotoxic chemotherapy and drug-eluting beads. However, no significant improvements were achieved regarding treatment outcomes 19. Until 2007, the number of patent publications peaked, when an oral multi-kinase inhibitor targeting vascular endothelial growth factor (VEGF), sorafenib from Bayer was approved by the US Food and Drug Administration (FDA) as the first line treatment for advanced HCC based on a 3-month median overall survival (OS) improvement compared with placebo (10.7 months vs 7.9 months; HR, 0.69) 20. Sorafenib, as the historically first effective systemic treatment option, paved the way for future innovations in molecular targeted therapy for advanced HCC. Whereas, the biggest limitation of sorafenib is the adverse events, including diarrhea, hand-foot skin reaction, weight-loss and hypophosphatemia 20. Thus, it is not uncommon to see that patients discontinue treatment due to poor tolerance and dose reduction. As a result of its toxicity profile and marginal efficacy, researches of novel targeted agents, such as brivanib, sunitinib, linifanib and lenvatinib, to compare with sorafenib were conducted in the decade after sorafenib was approved by FDA 11. But none of them have demonstrated superiority to sorafenib on the OS in clinical trials 11. While the HCC treatment patents numbers have dramatically increased during the last 10 years, the amount in 2016 reached the second peak, with 124 patent families filed in 266 patent documents. Major breakthroughs occurred since this year in management of advanced HCC. By 2016-2018, several tyrosine kinase inhibitors (TKI) as molecular target therapies have demonstrated clinical benefits, along with lenvatinib being approved as another 1st line therapy, as well as regorafenib, cabozantinib and ramucirumab as 2nd line therapies for advanced HCC 12. In the most recent 4 years, scientists or company industrial R&D spent their efforts mainly on immune checkpoint molecules as therapeutic targets and combination strategies 11. In 2019, atezolizumab in combination with bevacizumab have been approved as 1^st^ line therapy for advanced HCC, setting up the new gold standard for the coming years 21. Overall, patents publication trend in HCC therapeutic area from present study is aligned with milestones in management of HCC from 1981-2019 (Figure 1).

## Cutting-edge technologies

**Figure 2:**
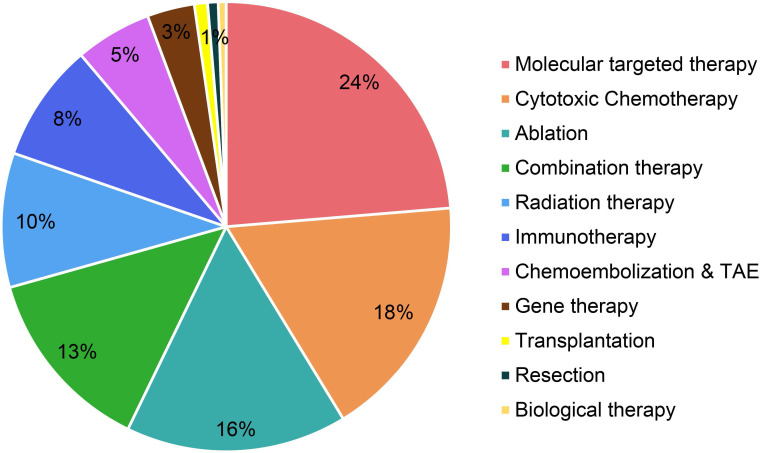
Static distribution of technological categories based on HCC patent family level.

The study retrieved patents samples with a priority date before December 31, 2019 using a series of searching terms related to HCC treatment in Derwent Innovation (https://clarivate.com/products/derwent-innovation/), a well-known patent database that is recognized worldwide. Data inclusion, screening and exclusion were conducted according to the rules of preferred reporting items for systematic reviews and meta-analyses (PRISMA) (Figure S1). To avoid missing data, the researchers searched these terms in diversified patent searching items, including the title and abstract. We excluded irrelevant patents by double-checking manually, and then used Derwent Innovation to deduplicate records. Technologies were classified in Figure 2 based on Barcelona Clinic Liver Cancer (BCLC) staging system and recent clinical publications 6,8,12,13. We marked technology category to patent records by a hierarchical reading order from title, abstract, claims, and full text.

### Molecular targeted therapy

**Figure 3:**
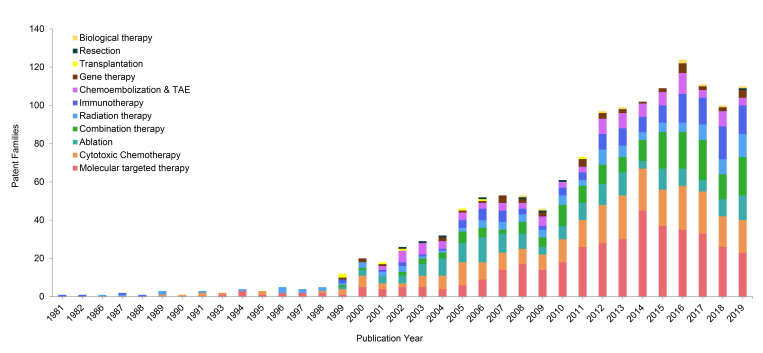
Annual changes of technological categories of patent families.

Molecular targeted therapy, as an emerging treatment modality for HCC, has been paid significant attentions in the technical invention. There are 126 patent families (filed in 743 patent documents) about molecular targeted therapy for treating HCC, accounting for 24% of all (Figure 2). Over the past decade, more than 20 patent families have constantly focused on molecular targeted therapy for treating advanced HCC in each year, and it peaked in 2014 with 45 inventions (Figure 3). After 2014, the number of patents related to targeted therapy is still stable, around 30 patents per year. As was discussed earlier, there were not many treatment options for advanced HCC, sorafenib was the only targeted therapy recommended on BCLC guideline to treat advanced HCC in 1990s 20. Nonetheless, in the wake of modest efficacy from sorafenib, there remained a critical and unmet need for aggressive development of innovative and more effective agents for advanced HCC. Therefore, it is not surprising to observe a large number of patents/researches dedicated to the development of targeted medications.

We have found a variety of mechanisms of these molecular targeted therapy from patent analysis, including carrying toxins directly to the cancer cell (WO2000053236), blocking the signaling pathway of cancer cell growth and division (WO2007041379), and changing protein activities within the cancer cell (WO2007075567). Some small molecules have been invented targeting factors involved in angiogenesis, such as VEGF (WO2013025944). Several drugs have been developed to inhibit epidermal growth factor receptor (EGFR) (WO2012087943), while others act on pathways that are existing targets of other drugs, such as mitogen active protein kinase (MEK) (JP06431770) and tyrosine-protein kinase Met (c-Met) (WO2007075567). Patent WO2007075567 described an invention to use triazolopyridazine compounds as protein tyrosine kinase modulators, particularly to inhibit c-Met activity and modulate c-Met expression in tumor cell in order to prevent tumor cell proliferation or disorder related to c-Met. As HCC has been shown with increased expression of hepatocyte growth factor receptor, the drugs that target to c-Met may have therapeutic efficacy against HCC tumor cells 22,23.

### Cytotoxic chemotherapy

Cytotoxic chemotherapy is the secondary hot spot in HCC patent research, which has 93 patent families (filed in 550 patent documents) being published, accounting for 18% of total patents (Figure 2). Traditionally, HCC is considered as a highly chemo-resistant tumor 24. Published clinical studies have shown poor results to treat HCC by employing conventional chemotherapy 24. However, our patent analysis has shown a growing interest about advanced forms of chemotherapy in treating HCC. Figure 3 shows the number of patent publications is increasing over the past decades. Different from conventional systemic chemotherapy, these inventions intended to improve efficacy and reduce toxicity via various technologies.

There are devices developed to remove chemotherapeutic agents (that are locally applied against solid tumor) from the blood coming from the tumor, in order to prevent contaminating the circulatory system (US4820261). Many patents claimed to use nanoparticle carriers loaded with anti-cancer agents to achieve more localized treatment (WO2008109163). In addition, back to 1995, electrochemotherapy was developed to introduce anti-cancer agents directly into cancer cells by increasing the permeability of cell walls through the use of electric pulses (US5468223). This technique is also called irreversible electroporation (IRE), which was further developed by Scherman Yves Leon as an individual from France in 2005, who used two electrode needles with two physical forces to enhance the penetration of anti-cancer agents into tumor cells (FR2872055). Hepatic artery infusion chemotherapy (HAIC) for local delivery of therapeutic agent on treating liver cancer was filed for patent by Abbott Cardiovascular Systems (WO2014117075) in the US, Japan, China and Europe from 2014-2016. HAIC is expected to provide a stronger antitumor effect and lower incidence of systemic adverse events due to the increased local concentrations in the tumor and reduced systemic distribution of anticancer drugs compared to traditional systemic chemotherapy 25. On clinical setting, HAIC has been used heavily in Japan for localized advanced HCC 24. However, there is no randomized controlled trial to provide evidence of survival benefits, thus no consensus on its place as a standard treatment for advanced HCC has been established.

In this study, patents in terms of cytotoxic chemotherapy also cover the use of doxorubicin (WO2000042832), method to shield bone marrow during chemotherapy (WO1999038442), stent as an intra-lumenal drug delivery device (WO2003103743), and thermoactivated drugs release (WO2006102471). Therefore, the growing interest of novel forms of chemotherapy for the treatment of HCC is mainly due to the technological advances that allow a targeted release of higher concentrated anticancer drugs.

### Locoregional therapy

For HCC patients with cirrhosis who are not eligible for surgical therapies, locoregional therapy provides minimally invasive procedures, including ablation, bland embolization, TACE, trans artery embolization (TAE), DEB-TACE and radiation therapy, and these technologies have been filed for patents, accounting 29% of all in this study (Figure 2). These techniques are performed under imaging guidance, and the maximum efficiency can reach to up to 80% as complete response 12.

Assorted types of ablation technologies have been discovered in this patent analysis, containing radiofrequency ablation (RFA) (WO2004067015, US20100168571, WO2019147185), microwave ablation (MWA) (CN1541628, CN1496276, CN1676176, WO2011063061, WO2016197093, JP2018114299), high-intensity focused ultrasound (HIFU) (US20060206105, WO2007112578, US20080200806, WO2010127495, US20110034833, WO2011069985, WO2011156624, KR2012117510, US20150352379), electroablation (WO2004110371, WO2005065284, WO2011081996, WO2012071526, WO2013091657, US20150320480, WO2016089781, US20160287313, WO2016201264, US20180036529, US20180125565, WO2018224404, US20190099214, WO2019185331) and laser ablation (US20030097152). Among them, electroablation has gained the most researchers' attentions.

In general, RFA is the most commonly used and preferred ablation treatment for HCC, which delivers electromagnetic pulse to cause tumor necrosis by heat injury 26. Percutaneous ethanol injection (PEI) is the other one of the most common chemical ablation techniques for HCC patients by using ethanol as cytotoxic material to lead tumorous tissue necrosis, tumor microcirculation and resultant ischemia 27. Because of the robust complete response rate (90-100%) and minor side effects in only about 5% of patient population, RFA and PEI have been recommended as curative therapy for early stage HCC on BCLC guideline 6. In the last 15 years, around 10 patents in terms of ablation have been published each year, and the gravity of innovations was more towards MWA and electroablation. MWA has several advantages over RFA, such as higher temperature range, shorter duration of procedure and less risk of skin burning 13. However, randomized controlled trials for comparing RFA vs. MWA showed no significant difference on efficacy for treating HCC patients 28. Regarding electroablation, it applies electrical pulses to tissue in a manner which destroys cancerous cells while sparing healthy tissue 29. Electroablation caught the most of attentions on optimizing the techniques in this study comparing with other ablation methods. As the healthy tissues in proximity to the destroyed cancer tissue by electroablation can be preserved, this technique is able to delineate the tumor boarders with short duration of procedure 29-31. However, it is less commonly used in clinical practice at the moment due to the lack of clinical evidence.

Although TACE and TAE have been widely used on HCC clinical setting, their total patent families only accounts 5% of all inventions (Figure 2). They constantly contribute around 5 patent families each year since 2001 (Figure 3).

Conventional TACE involves the injection of selected chemotherapeutic agent mixed with lipiodol (a contrast medium made from poppy oil), followed by injection of embolic material via the hepatic artery 32. TAE known as bland embolization, refers to as the embolization of the hepatic artery without using any chemotherapeutic agents 33. TACE has more than 30 years history, tracing back to the innovation of transcatheter technology in 1953 and application to unresectable HCC in 1980 32. Our research time scale is from 1981 to 2019, thus the patents before 1981 are missing. As early as 1974, embolization of hepatic artery to treat malignant liver tumors was reported by Doyon et al in France 34. In 1977, Yamada et al in Japan performed the first TACE, thus Japan has the longest history of using TACE, and numerous important technical developments of TACE are from Japan 35. Therefore, conventional TACE technology established since four decades ago are currently very mature with a rather stable market in treating HCC nowadays. This might be the reason of small proportion of TACE inventions existing in the present study.

The first patent related to TACE in our study was filed in 2001, discussing about catheter and perfusion system in arteries (WO2001003755). After that, the majority of patents were concentrated on DEB-TACE, using different types of materials as a carrier to deliver anticancer agent to tumor as well as to embolize the tumor blood supplies, and provide a localized drug release to the tumor. Based on our patent dataset, these carriers/beads could be made of hydrogel (WO2009073193), silicon (WO2002067998), magnetic nanoparticles and embolism composite (CN102652729), polymers (WO2004071495), polyvinyl alcohol nano-fiber particles (CN108187127) or gelatin sponges (WO2016093412). The growing interest on DEB-TACE may be due to the limitation of conventional TACE, which doesn't have a standardized protocol about the choice, dosage, concentration, rate of injection of chemo drugs, and optimal retreatment strategy 32. The argument over DEB-TACE vs TACE goes on and on during the past decade. Those who support DEB-TACE reported that DEB-TACE is using more reliable carriers that can increase the treatment efficacy and reduce the toxicity, and the standard protocol of DEB-TACE can make the procedure repeatable and reproducible 36. On the other hand, many investigators especially those in Asia led by Japanese physicians suggested that conventional TACE overperforms DEB-TACE, as lipiodol in the form of liquid different from solid particles can provide a better penetration into tumor capillaries 37. Nevertheless, more and more current inventions have attempted to optimize the technology based on DEB-TACE by making the beads to be in precisely controlled smaller sizes to enhance penetration (CN108187127), as well as developing radiopaque beads to provide visibility during and after the procedure (WO2005030268, WO2012101524, WO2015033093, CN105517580) and developing biodegradable beads to be less harmful to organs for the long term, as beads will stay permanently in patients' body (WO2015105459, US20130256928).

Radiotherapy is another local regional therapy that has been proven with promising therapeutic results. In fact, TACE and ablation have their own limitations. For instance, HCC with portal vein thrombosis (PVT) is a contraindication to TACE, while HCC with bleeding diathesis is a contraindication to RFA and MWA 38,39. Ablation is also very limited with tumor locations. For instance, it is difficult to ablate tumors that are closed to major bile ducts, pancreas and diaphragm 40. Radiotherapy including both external beam radiation therapy (EBRT) and selective internal radiation therapy (SIRT), accounts 10% in this distribution (Figure 2). The patent publications of radiation therapy are showing an increasing tendency during the past few decades (Figure 3). The role of radiation therapy in management of HCC has developed into more precision local therapy, attributed to the improved imaging technology and the advancement of radiation platforms 41.

Historically, conventional EBRT has had a limited role in treating HCC because there is 8.4% risk of causing radiation-induced liver disease (RILD) when the radiation dose is higher than 50 Gy 42. In general, >50 Gy radiation dose is necessary to achieve 77% rate of tumor response 43. Therefore, EBRT faces a challenge on balancing between the efficacy and toxicity. The patent analysis has shown that tremendous efforts have been made by researchers/scientists on improving the EBRT technique by applying alternative absorption frequency or radiation beam (WO1989010158, US4815448, WO2007067830), aiming to protect non-target cells from radiation by employing radioprotectants and fibrous spacers (WO1994022484, WO2017095788, WO1997016221, KR2012095917), to optimize dosimetry (WO1996034632, CN101518670, US9555264), and to enhance radio sensitivity (US20120315320, WO2016112268, US20170319692, WO2018134443). Imaging localization or accurate alignment (JP2005230561) during procedure is very critical in EBRT technique. In order to be a more focal radiation approach, ultrasound image-guided tissue-damaging procedure was developed in 2006 (WO2006018837), and *in vivo* imaging targeting the enzyme aldehyde dehydrogenase was invented in 2010 (WO2010048144). Charged particle therapy is an advanced technique of EBRT to overcome dosimetry challenges, such as radium particle therapy (EP1644049), neutron capture enhanced particle therapy (NCEPT/NCT) (WO2013003343, US20130090513, US20140179978, US20170216631), and radioactive nanoparticles for NCT (WO2016191247, WO2017096342, WO2019204645).

Other than the approach to give radiation from outside to inside patient's body like EBRT, scientists/researchers have also invented SIRT to offer radiation from inside in a localized manner. SIRT in treating HCC involves the injection of microspheres with radioisotopes or radioactive complex through hepatic arteries, which supply more than 80% of the total blood to the tumor 44. Upon trans-arterial administration, these microspheres get lodged in the tumor capillaries and release radiation energy locally, thus most of the normal parenchyma is preserved 44. Different from EBRT, this approach can give much higher dose, e.g. around 150 Gy, to the tumor, which was established as the effective dose threshold of treating HCC 45. However, there is no clinical evidence to show the superiority of SIRT over EBRT in terms of OS rate, and it quite depends on a proper patient selection. With these potential advantages of SIRT, scientists developed radioactive chitosan complex (US5762903), polymer as vehicle delivering radionuclides (US5942209), glass microspheres (WO2002034298), strontium-phosphate microparticles (US20150118495, WO2016064379), iodine-131 carbon microsphere (CN106178006) and resin microspheres (WO2019222700). Among these inventions, the most common clinically used SIRT is glass or resin microspheres labelled with Yttrium-90 46.

In recent years, there are increased clinical evidences of efficacy of radiotherapy in HCC either used alone or in combination with other therapies 46, and several HCC treatment guidelines have included radiation therapy as a treatment option for unresectable HCC, such as Asian Pacific Association for the Study of the Liver guideline 47, the Korean Liver Cancer Association - National Cancer Center guideline 48, Chinese guideline 49, and National Comprehensive Cancer Network guideline 50.

### Immunotherapy

Immunotherapy alone occupies 8% of the total patent families, excluding the ones being claimed as combination therapy (Figure 2). The number of patents in immunotherapy dramatically increased in recent years (Figure 3), and such booming in this technology especially since James Allison and Tasuku Honjo were awarded the 2018 Nobel Prize in physiology or medicine, due to their breakthrough work in immunology cancer research 51.

HCC is an immunogenic cancer, because it is usually developed from chronic liver hepatitis accompanied with liver cirrhosis, due to viral or non-viral pathogenesis. Such inflammation is associated with high tumor immunogenicity 52,53. Therefore, immunotherapy has been considered an effective treatment approach for HCC. Scientists/researchers have spent lots of efforts to support this hypothesis. However, as liver itself plays a critical role in host defense and self-tolerance, there is a variety of mechanisms underlying HCC tumor microenvironment, including immune suppression, immune evasion, effector T-cell dysfunction, low expression of tumor antigens (leading to low T-cell activation), cytokine deregulation and alteration in immune checkpoint molecules expression 52. Any one of these underlying mechanisms could be a strong obstacle to achieving an effective immune response to counteract tumor via immunotherapy. In this regards, immunotherapeutic strategies have been invented to act against these intrinsic immunologic characteristics of HCC tumor microenvironment.

Thus far, two checkpoint inhibitors have been developed, which are cytotoxic T-lymphocyte-associated protein 4 (CTLA-4) inhibitors and programmed cell death protein 1 pathway (PD-1/PD-L1) inhibitors. Accordingly, human anti-CTLA-4 antibodies (US20030086930), human antibodies to PD-1 (WO2015112800, US20180022809) and anti-PD-L1 antibody (WO2016061142, US20180186882) were found in our patent dataset.

Since HCC tumor expresses insufficient antigens to activate T-cells, the first immunotherapy patent in our dataset is from Japan dated back to 1999, which is about replicating defect recombinant retrovirus containing a vector structure to command the antigen expression (JP11262397). In 2016/17, new technologies were developed to deliver an antigen to the cytosol of an immune cell (WO2016070136), and intratumorally deliver particles containing a tumor antigen (WO2017181128). Furthermore, many works intending to activate and regulate T-cells have been done, for instance, using recombinant replication-deficient cytomegalovirus to generate a long-term, repeatedly stimulated T cell-based immune response (WO2011093858), generating antigen-specific CD8+ regulatory T cells (WO2012149282), converting the negative signal of TGFβ for T cell proliferation into a T cell activation signal (WO2014172584), promoting the formation, expansion and recruitment of T-cells in an antigen-specific manner (WO2016198932), using antigen-presenting cell-mimetic scaffolds to manipulate T-cells (WO2018013797), regulating T-cells production by nano-pulse stimulation (WO2018106672) and disrupting tumor microenvironment to regulate (chimeric antigen receptor) CAR-T cells (US20190287656). Additionally, in order to counteract cytokine dysregulation in HCC, inventors have also worked on modifying cytokines as anti-tumor medicine (CN1853730), producing cytokine in a cell to increase tumor specific immune response (CN102264760) and preparing tetrameric cytokines with improved pharmacokinetics by the dock-and-lock technology (US20130109073).

Currently, clinical exploration on the use of monotherapy with immuno-oncologic agents in HCC treatment is on the rise. Two anti-PD-1 antibodies to treat advanced HCC have been approved by FDA so far, which are nivolumab and pembrolizumab 54. Several combination regiments, e.g. anit-PD-1/PD-L1 antibody plus anti-CTLA 4 antibody, have been applied in clinical settings as well, which will be discussed in the next section. It is noteworthy that immune checkpoint inhibitors may cause serious toxicities 55. Different from other cancer patients, management of such toxicities in HCC patients could be more challenging, as HCC patients usually suffer from advanced chronic liver disease 56. Although we have not seen any technology development on reducing potential toxicities of immunotherapy from this patent analysis, it could be a future focus in immunotherapy research for safely treating HCC.

### Gene therapy

Gene therapy is defined as curing a genetic condition by delivering therapeutic nucleic acids into human cells, which can be performed either *in vivo* or in *ex vivo* 57. In terms of treating cancer, scientists developed multiple methods to fix the mutant or abnormal gene expression. Back in 1999 to 2004, gene therapy research was focused on identification of the novel role of a specific gene in cell transformation and tumor cell proliferation, and delivery of nucleic acids that can inhibit this specific gene expression or inhibit of this gene in transformed cells in order to reverse the transformed phenotype (WO1999055380, WO2000029589, WO2004009112). Suicide gene therapy is another approach to treat cancer by activating or introducing suicide genes that produce molecules causing cancer cells to kill themselves via apoptosis (WO2007036233). Another major gene therapy approach is introducing a small interfering RNA (siRNA) to inhibit oncogenic mRNAs translation (US20160168573).

In the area of gene therapy, the most challenging and important research question in the past decades was that what delivery systems should be used to introduce therapeutic nucleic acids, siRNA or suicide genes. In 2011, the invention (WO2011043719) provided replicating-competent adenoviral vector systems carrying one or more inserted heterologous gene that could offer enhanced transfection efficiency and specificity for gene delivery. Adenoviral vector is an efficient system for HCC hepatocyte targeted gene therapy by affecting non-dividing hepatocytes, because liver normally has less than 1% of dividing hepatocytes 57. Regarding *in vivo* gene therapy, the risk of adverse effects is high, as nucleic acids are directly delivered into patients' body through intravenous, intra-arterial, intra-portal and intra-tumoral administration 58. Nanoparticles as a non-viral delivery system can reduce the risk due to targeted delivery into cancer site via enhanced permeability and retention (EPR) effects 58. Therefore, nanoparticle used for intracellular gene delivery has become a research and development hotspot in recent years (WO2015042101, WO2017139758, WO2019213308). Cells can take up nanoparticles by endocytosis. Subsequently, nucleic acids such as siRNA will be released from nanoparticles to target mRNA degradation and inhibit RNA transformation. There are some studies about using nanoparticles as gene delivery carriers to treat HCC with HCC cell lines or animal models, however these studies have not moved into clinical trials yet 57.

The technology development pace in the field of HCC therapeutics has not been as much as with other cancers, although huge milestones have been made in gene therapy during the past decades. However, gene therapy has developed in a much slower pace, when compared to molecular targeted therapy (Figure 3), with only 3% contribution in HCC patents (Figure 2), which may be explained by the ethical questions surrounding gene therapy. Although researchers are attempting to find out targeted gene therapy for HCC, it is difficult to determine a single target gene, because HCC is polygenic and multifactorial. Therefore, combination therapy might be more effective than standalone therapy.

### Combination therapy

Regarding all aforementioned HCC therapeutic options, there may be a ceiling of effects for using monotherapy, especially for advanced HCC, as for cancer treatment, there is no such “one fits all” solution. Thus, combination therapy became a hotspot to enhance the treatment efficacy. In the present study, the amount of patent publications related to combination therapy accounts for 13% of all HCC treatment patents (Figure 2). During the past 20 years, the attentions from researchers on combination therapy for HCC have significantly increased (Figure 3).

In this study, around 46% of combination therapies are about combining immunotherapy with other types of therapy as immunomodulatory approaches. The basic mechanism behind is to positively modulate the tumor microenvironment (TME) aiming at counterbalancing the strong immune suppressive setting. Among them, this present study shows that the research gravity is moving more towards molecular targeted therapy in combination with immunotherapy, such as antibodies binding to human CSF-1R plus immunotherapy (CN104271158), mTOR inhibitor plus anti-CD20 antibody (JP06042801), anti-IL-10 antibody or antigen binding fragment plus CpG-C oligonucleotide (CN107949399), and anti-PD-L antibody plus DNA-PK inhibitor (WO2018178040). This trend is aligning with the hotspot in clinical research, as recent clinical studies have demonstrated the more potent anti-tumor effects by combination of an anti-PD-1 antibody and an anti-angiogenesis agent 59. Accordingly, in May 2020, FDA approved the use of atezolizumab (immune checkpoint inhibitor) in combination with bevacizumab (antibody against VEGF) for unresectable HCC 59.

Radiotherapy is used for unresectable HCC, which can induce tumor apoptosis and necrosis, as well as releasing tumor associated antigens and neoantigens at the same time 60. In this way, radiation therapy can significantly induce intratumoral immune infiltration and immune response activation 60. Therefore, combination of radiation therapy and immunotherapy have been investigated and the resulting technology has been filed for patents, such as radiation therapy plus antigen presenting cells (WO2014066615), radiation therapy plus PD-1 and/or PD-L1 antagonist (WO2015193352), and check point inhibitor (inhibits interaction of PD-1 and PD-L1) plus radiation therapy (US20190160179). Furthermore, there are several other inventions to increase cancer antigen release and to enhance the immune responses, for instance, chemotherapy (US20140186375), thermotherapy (US20190091350), and by using peptide-protein conjugate (WO2016041014), immunomodulatory polynucleotide (WO2017201325) or fusion protein of Flt3L and albumin (US20190209649). Besides modulating tumor antigen expression and TME, there are multiple mechanisms of chemotherapy to enhance antitumor immunity, including selectively killing immunosuppressive cells, induction of immunogenic cancer cell death and modulating immune checkpoint molecules 61-63. Recently, locoregional therapy is also considered as a potent option to have synergistic action with immune drug, because locoregional therapy such as TACE and ablation can cause immune reaction against the tumor. Duffy et al. 2017 reported that RFA and TACE could enhance the efficacy of Tremelimumab (anti-CTLA-4) 64. However, we have not seen any patent in this analysis related to such combination regimens. In a near future, it will be interesting to explore the possible synergy and combination between standard treatments and immunotherapy.

Despite the enhancement of immune response, there are also abundant other combination regimens showing in the patent database, such as chemotherapy plus radiotherapy (WO2015109367, US20150315131, WO2016011328, US10456468), molecular targeted therapy plus TACE/TARE (WO2015123818, WO2019015561), RFA plus cancer drugs (WO2016015015, WO2017214974), miRNA plus radiation/chemotherapy (WO2016149580), hyperbaric oxygen with histone deacetylase inhibitors plus glycolytic therapies (US20150090267, US20170007573) and alcohol ablation plus ultrasound (CN110101859). Altogether, emerging strategies with novel agents combing with the existing therapeutic approaches bring higher possibility to improve HCC patients' therapeutic outcomes.

### Others

Patents related to transplantation, resection and biological therapy have the smallest proportions, less than 3% in terms of each type of therapy (Figure 2). Although resection and transplantation are the two major options in curative setting for early stage HCC patients, we are not surprised to see such a small amount of related patents, as they are surgery based, and not many advances on technologies can be developed in comparison with other treatment modalities. The regarding technologies filed in patents landed on: image display device for accurate resection (CN110310726), isolating normal hepatocytes from unwanted cells by magnetic separation (KR2006067974), preventing rejection of transplanted organs (US6970741, US20060036286, CN102274088), and inhibiting damage of donor tissue (WO1999005989, WO1999029306). Of an important side note, resection is more aggressively conducted in Asia, even being applied on advanced HCC patients with PVT, while resection is mainly used for early stage patients in the western countries 65. On the other hand, transplantation is very limited in Asia 65. Nevertheless, resection has been frequently used to effectively treat HCC patients worldwide, and recent strategies of downstaging or bridging more patients to transplantation by locoregional therapy brought significant benefits to HCC patients 66,67.

Figure 3 also shows that the patent publications in biological therapy began to gradually rise after 2000. Biological therapy is considered as a type of treatment using substances from living organisms to treat disease, for instance, stem cells (WO2008060788), monocyte derived cell infected with an oncolytic herpes simplex virus (WO2016146535) or an oncolytic virus (JP2017171668) in treatment of HCC. The staggering concept of biological therapy in HCC is still in the infantile stages of development.

## Therapeutic technology flow

In many studies, patent citations have been employed to explore technology transfer and technology flow 68. Network topological analyses could help us to improve understanding of the technology diffusion process by network statistics to characterize the structure of large-scale networks 69. In the present study, a patent citation network analysis was carried out to conduct the critical node analyses and topological analyses in order to identify and understand HCC therapeutic technology flow. The nodes from the citation network were distributed using the Fruchterman Reingold layout following manual adjustment, which is a force-directed layout algorithm. The clusters in the network were detected using the Louvain modularity method 70.

**Figure 4:**
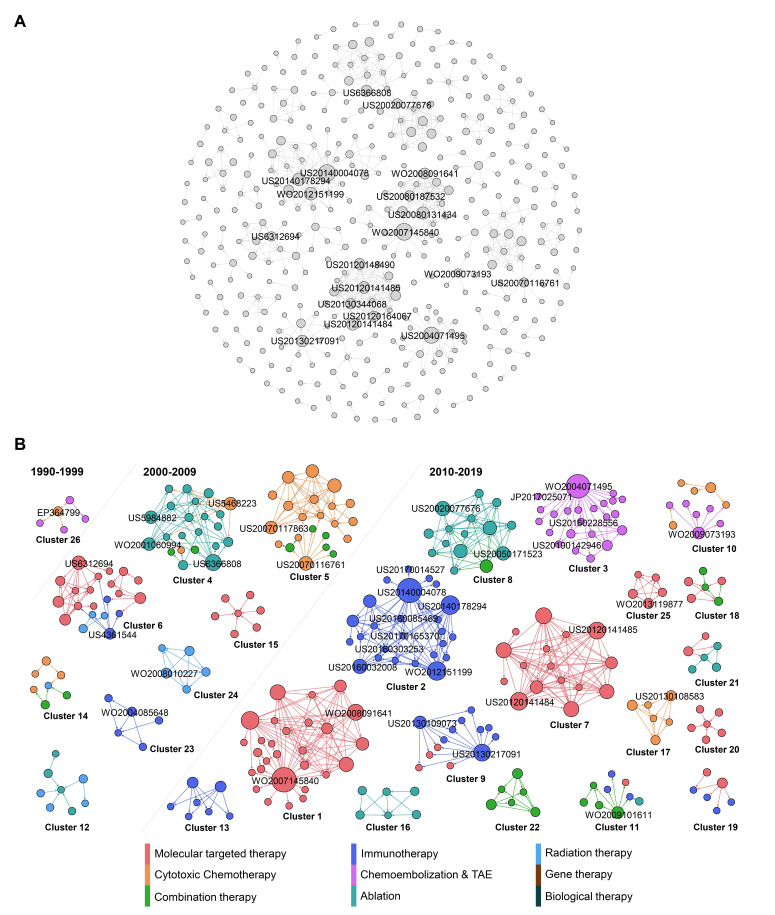
Citation analysis of HCC treatment patents. (A) Global citation network including all patents and their citation links. Bigger nodes represent highly cited patents. The node size was set according to its out-degree value, that is, the greater the out-degree, the larger the node size, and the more citations a given patent received. (B) The isolated cluster of the patent citation network showing different communities identified by the metrics of modularity. Patents are classified into 9 types, each with a different colour. The cluster numbers are ordered by node and edge ranking from the highest to lowest within the cluster network. The nodes are coloured based on the classification while the edges are coloured based on the source of the citation.

Figure 4A reveals the citation network of HCC treatment patents. The nodes represent patents, and arrows represent citation connections between cited and citing patents in the network. In total, 402 nodes and 676 edges have been plotted in Figure 4A, which visualizes a landscape of HCC inventions by highlighting clusters and key inventions within clusters, while evolution process of these clusters are shown in Figure 4B. To highlight the main technology clusters, clusters with more than five patent members are marked in different colors. The largest community in brown color represents 6.97 % of the total nodes, the second in teal comprises 6.22 % of the total nodes, and the third in mint green takes 5.72% of the nodes. The brown community (cluster 1) includes patents with many big nodes, highlighting its importance to the network since it gathered the most frequent cited patents. Cluster 1 is highly interactive, presenting the on-going development of molecular targeted therapy. Cluster 2 in teal is purely dominated by HCC therapeutic option of immunotherapy. Cluster 3 in mint green is also dominated by a single type of therapy, TACE.

In general, this citation map shows that the major clusters do not have connections with each other, and they all belong to the type of technologically concentrated community. This is different from our previous studies 71-73, where patent citations represent much more interactions. This interesting discovery may be attributed to the unique, complicated and heterogeneous etiology of HCC leading to the needs of therapy diversification, utilizing distinctive technologies, which could barely have overlapping technology development. For example, radiation therapy using medical device and immunotherapy using drugs do not have any technology interactions, although they can be combined in clinical setting due to their synergistic effects.

In order to reflect the evolution of network clusters, the 26 clusters with more than five patents were extracted and divided into three time periods, 1990-1999, 2000-2009, and 2010-2019, as illustrated in Fig. 4b, based on the average year that the patents were published within a specific cluster. Here, the most cited patents are about molecular target therapy (Figure 4B). Overall, the number of patent clusters is dramatically increasing in the period of 2010-2019, when compared to the previous two periods. Back to 1990-1999, the HCC therapy investigation was only located on cytotoxic chemotherapy and TACE. During 2000-2009, while the exploration on cytotoxic chemotherapy was still active, many emerging therapies in HCC were developed, including ablation, immunotherapy, radiation therapy and combination therapy. Ablation and chemotherapy are the research protagonists in this period. Moving into 2010-2019, more active and diversified innovations have been achieved. We see the protagonist in HCC therapy has been changed to target therapy, immunotherapy and DEB-TACE.

When patents have a high out-degree in citation network, it means that these patents are mainly cited by subsequent patents, because they contain either fundamental or innovative technologies, which others in the field are trying to imitate 74. These influential patents with more citations are distributed in different clusters and are in the relative central position of the citation network, which played critical roles in technological flows, such as WO2007145840, US20140004078 and WO2004071495 in cluster 1, 2, 3 respectively.

WO2007145840 in cluster 1 is the invention that for the first time identified antibody that specifically binds to a non-ligand binding region of the extracellular domain of a human Notch receptor therapeutically effective against cancer. In the field of HCC therapy, research on signaling pathways are of interest because of their specific anti-cancer characteristics. For example, sorafenib is the approved therapy for advanced HCC by targeting ras/raf signaling pathway 20. Notch signaling pathway is another one of the traditional pathways considered to be critical in HCC management, as it involves the regulation of angiogenesis, cell differentiation, cell proliferation and survival. Several critical and emerging studies have linked Notch signaling to hepatogenesis and hepatic duct morphogenesis 75,76. Further role of Notch in liver development is demonstrated to be liver regeneration after injury by activating Notch pathway 77-79. More specifically, Notch 1 and Notch 2 isoforms are identified as parallel functional pathways during liver regeneration 80,81. Interesting alignment has been found in the invention of WO2008091641 in our study, which is also a big node representing an influential patent in cluster 1. This invention for the first time identified a conserved ligand binding region comprising a conserved glutamate within epidermal growth factor inhibitor 1 (EGFI l) of Notch 1, Notch2, and Notch4 and EGFI 0 of Notch3 specifically. Moreover, this invention provides isolated antibodies targeting to these Notch receptors in order to inhibit tumor growth.

All the patents in cluster 1 are related to Notch pathway belonging to OncoMed Pharmaceuticals, Inc. These inventions are the scientific basis of their two molecular targeting clinical pipelines, Tarextumab and Brontictuzumab that are being developed in collaboration with GlaxoSmithKline 82. Tarextumab is a human monoclonal antibody targeting Notch 2/3 receptors. It has been approved by the US FDA as an orphan drug for treatment of pancreatic cancer and lung cancer. Brontictuzumab is a human monoclonal antibody targeting Notch 1 for cancer treatment 83. However, they have not been officially approved by FDA for HCC treatment yet, although manipulation of Notch receptors has already been studied in HCC with novel and potential breakthrough into the role of Notch in HCC. Notch-based strategies will be investigated continually, and combination with other approved treatment approaches will provide alternative treatment options to aggressive cancer types, such as HCC 83.

In the second largest cluster 2, the most influential node represents patent of US20140004078, which is the invention of antibody drug conjugates (ADC) technology. The monoclonal antibody and therapeutic agent are linked by an acid cleavable linkage to increase cancer therapeutic efficacy (US20140004078). For many years, it has been a scientific goal in the field of specifically targeted drug therapy to use monoclonal antibodies for the specific delivery of toxic agents to human cancers. Conjugates of tumor-associated antibodies and suitable toxic agents have been developed but have had mixed success in the therapy of cancer. During the development of this ADC technology, the most critical challenge was believed to be the linker between the antibody and drug for retention of good anti-tumor activity. With that, inventors have developed novel linkers for a variety of drugs or cancer toxic agents, such as disulfide linkage and ester linkage 84. The acid cleavage linkage from a present invention (US20140004078) has been cited by many other immunotherapy inventions in cluster 2 (Fig. 4b), suggesting its importance and significance in the ADC field.

In addition, we have found another influential invention in cluster 2, which is a method using ultrafiltration and diafiltration to prepare stable and high concentrated formulations of immunoglobulins, antibodies and antigen binding antibody fragment (US20140178294, WO2012151199 in same patent family). While the standard mode of antibody administration is intravenous infusion, several side effects such as rash, hypotension or urticaria have limited the antibody infusion rate 85,86. In order to address this special issue, subcutaneous, intramuscular and transdermal administrations have been proposed, but they are all associated with lower injection volume 86. Thus, there is a need for more concentrated antibody.

Overall, abovementioned three biggest nodes in cluster 2 have highlighted the two essential elements in the field of using ADC to effectively treat cancer, which are AD linkage and antibody concentration. With these achievements, many specific technologies in this field have been invented by citing these two inventions. If we look into the small nodes with higher in-degree in cluster 2 (US20160303253, US20170165370, US20180085469 in same patent family) that have cited the biggest nodes, it is obvious that the technology flows to anti-Trop-2-SN-38 immunoconjugates with a CL2A linker with specified dosage to maximize efficacy for cancer treatment with minimal side effects. While anti-Trop-2 is the antibody, SN-38 is one of the potent camptothecin derivatives that is potent antitumor agent.

All the patents in cluster 2 belong to Immunomedics, which is a pharmaceutical company focusing on human monoclonal antibody and ADC for treatment of cancer. From cluster 2, we can see that antibody-SN-38 immunoconjugates are their focus in recent years, also could be their future R&D development direction. One of the clinical pipelines from Immunomedics, sacituzumab govitecan (IMMU-132) is an anti-Trop-2-SN-38 ADC for cancer. Phase I/II study of IMMU-132 in patients with epithelial cancers including HCC is undergoing now 87. In fact, IMMU-132 has just been approved for treatment of breast cancer in April 2020 by the US FDA 88. Moreover, based on the patent content that claims antibody-SN-38 immunoconjugates in combination with other therapeutic modalities in certain embodiments (US20170014527), we predict that combination use of this ADC may be carried on in a near future.

WO2004071495 in cluster 3 is the invention of polyvinyl alcohol-based polymer particles loaded with doxorubicin for the purpose of embolization of vessels supplying malignant hyper vascularized tumors, accompanied with a local, controlled, sustained delivery of doxorubicin to the tumors. This patent is the fundamental technology for the development of DEB-TACE that is one of the key clinical pipelines from Biocompatible UK company. This invention paved the way for DEB-TACE, compensating the limitations of conventional TACE, with numerous advantages including standardized procedure and reduced systemic exposure to doxorubicin. However, there is no head to head comparison in clinical trial to show if DEB-TACE overperforms cTACE in terms of HCC patients' survival.

From cluster 3, we can see that the DEB-TACE technology flows to imaginable radiopaque polymer (US20160228556, US20190142946) and beads in smaller size (JP2017025071). These patents are also from Biocompatible UK. Thus, it is not difficult to see that this company's strategy on DEB-TACE tends to make visible and smaller drug eluting beads. Several company sponsored clinical studies regarding to these new generations of DEB-TACE have also been published in recent years, including Lencioni et al 2018 and Aliberti et al 2017 89,90. Such R&D strategy is aligning with the recent clinical needs in the field of TACE. One need is delivering beads as distally and closely to the tumor as possible, being facilitated by the trend towards smaller micro-catheters which allowed a super selective approach 91. Logically, smaller beads are demanded as well. Another need for TACE is to see an accurate “footprint” of embolized vessels, which correlates with drug delivery, such that interventional radiologist is able to make a better decision about the endpoint during procedure, and to know which vessel is missing for embolization after procedure 91. Such finding is aligning with our previous discussion of TACE in technology characteristic session.

## Discussion

Based on the technology citation network, the major clusters do not have connections with each other, implicating a strong technology barrier among HCC therapeutic technology categories. This interesting discovery may be attributed to the unique, complicated and heterogeneous etiology of HCC leading to the needs of therapy diversification, utilizing distinctive technologies, which could barely have overlapping technology development. National borders have also been shown as a negative impact on knowledge flows. As geographical distance has a significant impact on knowledge spillovers 92, and cross-border cooperation could promote the development of technology innovation, knowledge spillover will be our future direction to study in depth.

Therefore, one assignee can't involve into multiple technologies. Company strategy teams can see a full picture of technology development trend and gain early insight into innovation competitors from this patent analysis. While combination therapy is one of the future trends in treating HCC, we recommend making investment onto advancement of combination therapy clinical evidence by cooperating with other companies, who have alternative therapeutic technologies. Such action also can break boundaries among various dispersed technology clusters in order to achieve the best clinical outcome in treating HCC. For big company with sufficient funds, acquisition and merge of other companies is an efficient strategy to integrate multiple technologies into one company, so that one stop window can be provided to HCC patients and related health care providers.

In addition, diversified HCC therapeutic modalities are recommended to get approval from government due to the heterogeneous HCC characteristics in order to achieve personalized therapy for individual patient. In the battle of fighting with this changeling disease, the emerging cutting-edge therapies are demanded to be covered by government reimbursement in order to fulfill affordability.

From researcher's perspective, molecular target therapy for advanced HCC was the dominant technology in concerned field with largest amount of inventions, which implicates the importance of exploring novel therapeutic targets and pathways by R&D teams. The trend of research and technology development in treating HCC tends to be more localized, penetrated, degradable (if using beads as drug carrier) and imageable therapies for the purpose of reducing toxicity and boosting efficacy at the same time, especially in chemotherapy and locoregional therapy developments, and there are increased clinical evidence on locoregional therapy nowadays leading the recommendations of it on HCC guidelines. Immunotherapy grows rapidly in the recent years, but it must be noted that there is a technology lacking on reducing potential toxicities of immunotherapy from this patient analysis, while it has been reported that immune checkpoint inhibitors may cause serious toxicities. Therefore, reducing such toxicities could be a future important focus in immunotherapy research for safely treating HCC. Regarding to gene therapy, research investment is recommended to be located at exploring delivery system, such as nanoparticle that demands clinical trials before using on HCC patients.

It is always difficult to determine a single target gene, because HCC is polygenic and multifactorial. Therefore, combination therapy might be more effective than standalone therapy. Investment and researches on studying emerging strategies with novel agents combing with the existing therapeutic approaches can bring higher possibility to improve HCC patients' therapeutic outcomes. We predict that the research gravity and company strategies will move towards molecular targeted therapy or other traditional therapies in combination with immunotherapy in the coming five years. Moving into 2010-2019, we see the protagonist in HCC therapy has been changed to target therapy, immunotherapy and DEB-TACE. Combination use of ADC may be carried on in a near future. Notch-based strategies will be investigated continually, and combination with other approved treatment approaches will provide alternative treatment options to aggressive cancer types, such as HCC.

This study still has some limitations. Some recent emerging but important patents may be disregarded when patents are analyzed in citation network, because latest patents are often cited less frequently. Thus, it is believed that continuous tracking of the inventions in the next several years remains the optimal approach to verify emerging favorable technologies. Another limitation is due to the nature of patent, where not that all R&D work on HCC therapy are fully reflected in patent-based analysis, although patent is generally considered as a key indicator of innovation and potential commercialization. In addition, many of the patents in this study are not specifically claimed for HCC, but also for other cancer types. This could be a potent cofounding factor in this research.

At present, there are a huge number of patents in the HCC field. This research mainly explores patents in the therapeutic field. The final patents included are designed to ensure accuracy. Noteworthy, there are enormous patents related to HCC, which include the relative surgery devices, fundamental experiments, biomarkers, vaccines etc. Although they cannot reflect HCC therapy development directly, they do indirectly contribute to the evolvement of HCC therapeutic field. In the future, we will continue to explore patent research in the entire related field. Overall, this HCC treatment patent landscape analysis still provides the first overview and significant supplementary knowledge about the previous and recent technological and clinical developments on HCC therapy.

By the end, this study is highly descriptive in nature by focusing on a specific therapeutic field. Future works are highly demanded from explanatory perspective by approaches of different statistical modeling techniques, such as special analysis, agent-based simulation etc. Furthermore, we are planning to add more attributes to transform this descriptive research into explanatory research or even predictive research.

## Supplementary Material

Supplementary figures.

## Abbreviations

ADC: Antibody drug conjugate
BCLC: Barcelona Clinic Liver Cancer
c-Met: Tyrosine-protein kinase Met
CTLA-4: Cytotoxic T-lymphocyte-associated protein 4
DEB-TACE: Drug eluting beads trans artery chemoembolization
DWPI: Derwent World Patents Index
EBRT: External beam radiation therapy
EGFI l: Epidermal growth factor inhibitor 1
EGFR: Epidermal growth factor receptor
EPR: Enhanced permeability and retention
FDA: US Food and Drug Administration
HAIC: Hepatic artery infusion chemotherapy
HBV: Hepatitis B virus
HCC: Hepatocellular carcinoma
HCV: Hepatitis C virus
HIFU: High-intensity focused ultrasound
IMMU-132: Immunomedics, sacituzumab govitecan
IRE: Irreversible electroporation
MEK: Mitogen active protein kinase
NCEPT/NCT: Neutron capture enhanced particle therapy
OS: Overall survival
PD-1/PD-L1: Programmed cell death protein 1 pathway
PEI: Percutaneous ethanol injection
PRISMA: Preferred reporting items for systematic reviews and meta-analyses
PVT: Portal vein thrombosis
R&D: Research and development
RFA: Radiofrequency ablation
RILD: Radiation-induced liver disease
siRNA: Small interfering RNA
SIRT: Selective internal radiation therapy
TACE: Trans artery chemoembolization
TACE: Trans arterial chemoembolization
TAE: Trans artery embolization
TKI: Tyrosine kinase inhibitors
TME: Tumor microenvironment
VEGF: Vascular endothelial growth factor
