# Supplementary material for: A review of cutting-edge therapies for hepatocellular carcinoma (HCC): Perspectives from patents
Source: Int J Med Sci. 2021 Jun 18;18(14):3066–81. doi: 10.7150/ijms.59930 (PMC8364461; doi:10.7150/ijms.59930)
Supplement: Supplementary file 1 — Supplementary figures. [file ijmsv18p3066s1.pdf]

SUPPLEMENTARY MATERIAL

Methods

Derwent Innovation platform was employed in this study to collect the sample data of patents related to HCC treatment worldwide, which were published before December 31, 2019. We searched keywords of ‘HCC’, ‘hepatocellular carcinoma’, ‘primary liver cancer’, ‘hepatoma’ contained in patent titles and abstracts. As the study focused on patents dealing with treatment or therapy, unrelated patents such as patents of biomarkers, diagnosis and vaccines were deleted manually. **Figure S1** shows the detailed process of data inclusion, screening and exclusion, according to the rules of preferred reporting items for systematic reviews and meta-analyses (PRISMA), which is an evidence-based minimum set of items for reporting in systematic reviews and meta-analyses [1].

The patent analysis in this study focuses on family-based patent information. The same invention disclosed by a common inventor(s) and filed in more than one country/region or patent office is called a patent family. The extended patent family was used in the study for counting inventions. An extended patent family was defined by International Patent Documentation Center (INPADOC) as a collection of patent documents covering a technology in which members of an extended patent family will have at least one priority in common with at least one other member, either directly or indirectly [2]. For all sampling patent records, this research selected various data, including inventor, assignee, the address of the inventor, the address of the assignee, international patent classification (IPC), citation information, application year and publication year. The items reported in this patent landscape study are according to the Reporting Items for Patent Landscapes (RIPL) checklist, which is the latest comprehensive standard for patent landscape reporting [3].

The method of network analysis in Figure S2 was applied to review HCC treatment patents to

highlight the citation relationship between patents. We constructed a series of patent citation networks, where nodes represent patents, and directed edges and arrows denote citation relationships and directions. Moreover, patents can be clustered together to form an independent network component, in which nodes have relatively frequent internal connections. The structures and features within patent citation networks comprehensively indicate the patterns of technology flows and evolution. Gephi software was adopted to visualize the network layout in this study. It is an open source and free cross-platform JVM-based network analysis software, which is mainly used for interactive visualization in various networks, complex systems, and layered graphs [4].

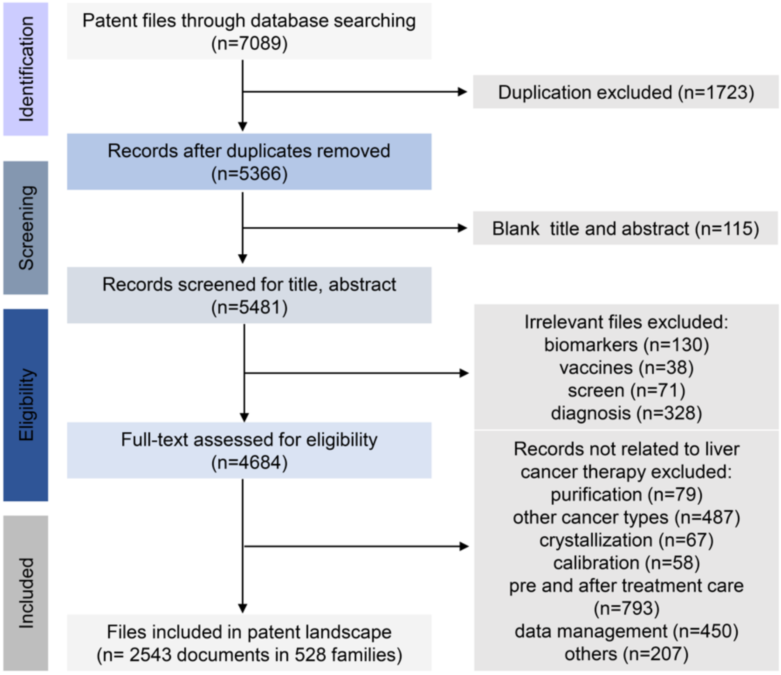

Figure S1. PRISMA flow diagram detailing the number of patent documents included at each stage and the reasons for removal.

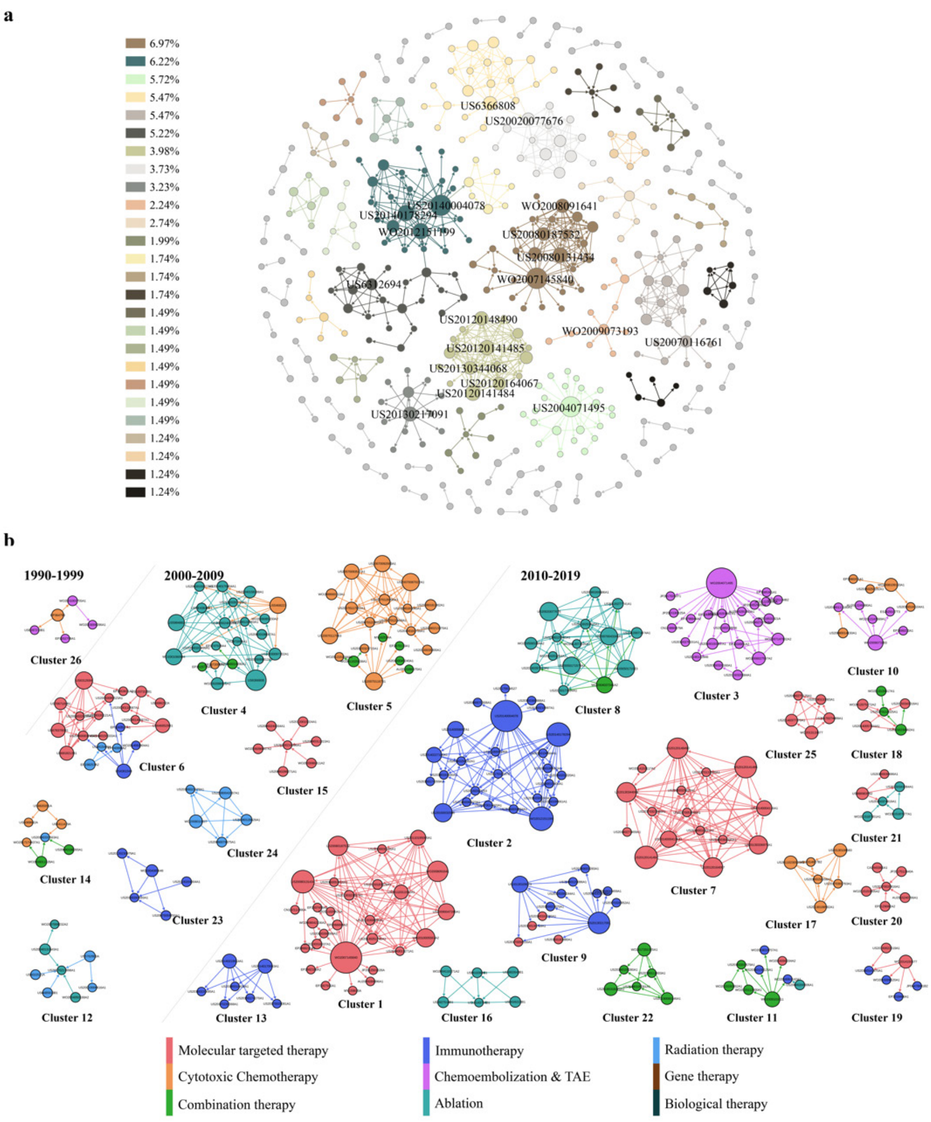

Figure S2. Citation analysis of HCC treatment patents. (A) Global citation network including all patents and their citation links. Bigger nodes represent highly cited patents. The node size was set according to its out-degree value, that is, the greater the out-degree, the larger the node size, and the more citations a given patent received. (B) The isolated cluster of the patent citation network showing different communities identified by the metrics of modularity. Patents are classified into 9 types, each with a different color. The cluster numbers are ordered by node and edge ranking from the highest to lowest within the cluster network. The nodes are colored based on the classification while the edges are colored based on the source of the citation.

References

[1] Hutton B, Salanti G, Caldwell, DM, Chaimani A, Schmid CH, Cameron C. et al. The prisma extension statement for reporting of systematic reviews incorporating network meta-analyses of health care interventions: checklist and explanations. Ann. Intern. Med. 2015; 162: 777-784.

[2] European Patent Office: INPADOC extended patent family. https://www.epo.org/searching-for-patents/helpful-resources/first-time-here/patent-families/inpadoc.html

[3] Smith JA, Arshad Z, Trippe A, Collins GS, Brindley DA, Carr AJ. The reporting items for patent landscapes statement. Nat. Biotechnol. 2018; 36: 1043-1047.

[4] Hanneman RA, Riddle M. Introduction to social network methods. California, USA: University of California Publisher; 2005.
